# Comparison of capability and health-related quality of life instruments in capturing aspects of mental well-being in people with schizophrenia and depression

**DOI:** 10.1192/bjo.2022.514

**Published:** 2022-06-27

**Authors:** Timea Mariann Helter, Joanna Coast, Agata Łaszewska, Tanja Stamm, Judit Simon

**Affiliations:** Department of Health Economics, Center for Public Health, Medical University of Vienna, Austria; Health Economics Bristol, Population Health Sciences, Bristol Medical School, University of Bristol, UK; Department of Health Economics, Center for Public Health, Medical University of Vienna, Austria; Section for Outcomes Research, Center for Medical Statistics, Informatics, and Intelligent Systems, Medical University of Vienna, Austria; and Ludwig Boltzmann Institute for Arthritis and Rehabilitation, Vienna, Austria; Department of Health Economics, Center for Public Health, Medical University of Vienna, Austria; and Department of Psychiatry, University of Oxford, Warneford Hospital, Oxford, UK

**Keywords:** Capability approach, EQ-5D-5L, ICECAP-A, OxCAP-MH, schizophrenia

## Abstract

**Background:**

There is increasing evidence that assessing outcomes in terms of capability provides information beyond that of health-related quality of life (HRQoL) for outcome evaluation in mental health research and clinical practice.

**Aims:**

To assess similarities and differences in the measurement properties of the ICECAP-A capability measure and Oxford Capabilities Questionnaire for Mental Health (OxCAP-MH) in people with schizophrenia experiencing depression, and compare these measurement properties with those of (a) the EuroQol EQ-5D-5L and EuroQol Visual Analogue Scale (EQ-VAS) and (b) mental health-specific (disease-specific) measures.

**Method:**

Using data for 100 patients from the UK, measurement properties were compared using correlation analyses, Bland–Altman plots and exploratory factor analysis. Responsiveness was assessed by defining groups who worsened, improved or remained unchanged, based on whether there was a clinically meaningful change in the instrument scores between baseline and 9-month follow-up assessments.

**Results:**

The two capability instruments had stronger convergent validity with each other (Spearman's rho = 0.677) than with the HRQoL (rho = 0.354–0.431) or the mental health-specific (rho = 0.481–0.718) instruments. The OxCAP-MH tended to have stronger correlations with mental health-specific instruments than the ICECAP-A, whereas the ICECAP-A had slightly stronger correlation with the EQ-VAS. Change scores on the capability instruments correlated weakly with change scores on the HRQoL scales (rho = 0.131–0.269), but moderately with those on mental health-specific instruments for the ICECAP-A (rho = 0.355–0.451) and moderately/strongly on the OxCAP-MH (rho = 0.437–0.557).

**Conclusions:**

Assessing outcomes in terms of capabilities for people with schizophrenia and depression provided more relevant, mental health-specific information than the EQ-5D-5L or the EQ-VAS. The ICECAP-A and the OxCAP-MH demonstrated similar psychometric properties, but the OxCAP-MH was more correlated with disease-specific instruments.

The capability approach is a theoretical framework with a range of potential applications. It has been highlighted as a way of helping people with mental health difficulties to engage with their values and priorities, for instance through influencing the design and delivery of mental health services.^1^ The capability approach was developed by Amartya Sen with a core focus on what individuals are free and able to do and be (i.e. are capable of).^1^ This approach places emphasis on promoting well-being through enabling people to realise their capabilities and engage in behaviours that they value.^1^

Economic evaluations are comparative analyses of alternative courses of action based on the costs and consequences of the alternatives being considered.^2^ There is increasing interest in the use of the capability approach for the economic evaluation of health-related interventions, including those in mental healthcare.^3^ One reason for this is the wider evaluative space this approach offers for the measurement of well-being in comparison to the commonly used methods of assessing effects in terms of health-related quality of life (HRQoL).^4^ Currently the EuroQol EQ-5D instruments (including a self-rated health assessment recorded on a vertical visual analogue scale, the EQ-VAS) are the most commonly recommended and used HRQoL instruments for economic evaluations in healthcare.^5^ However, HRQoL instruments such as the EQ-5D may not capture important effects of interventions when these go beyond health change and therefore underestimate their full effect on welfare.^6^ Mental healthcare interventions and services usually target both health and social impairments because many people with severe and enduring mental illness experience significant functional and social challenges which may also spill over into effects on education, employment, justice and families.^4^ There is evidence of these impacts beyond HRQoL in mental illness, and particularly in the case of schizophrenia.^7^ People with schizophrenia are more likely to be homeless, unemployed or living in poverty compared with the general population; moreover, their disease is progressive and causes increasing disability, dependence on care and need for assistance from others in carrying out activities of daily life.^8^ The prevalence of depressive disorder in schizophrenia has been reported to be around 50%.^9^ Evidence suggests that depression is linked to poorer outcomes in schizophrenia, as well as to particularly high levels of healthcare use and suicide.^10^

A recent literature review of capability instruments in economic evaluations of health-related interventions identified 14 such instruments, which differ substantially in their development methods, items, item levels, target populations and the interventions that were evaluated using them.^3^ Two of the most commonly used and validated instruments, particularly for economic evaluations among adults with mental health problems,^3^ are the ICECAP capability measure for adults (ICECAP-A) and the Oxford Capabilities Questionnaire for Mental Health (OxCAP-MH). Although both instruments are grounded in the capability approach, their conceptual approaches differ. The ICECAP-A belongs to a broader group of ICECAP capability instruments, each focusing on important capabilities according to a particular life stage.^11^ It was developed in the UK, using bottom-up participatory methods as recommended by Sen^12^ to generate contextual capability attributes for the whole adult population. The OxCAP-MH was also developed in the UK, originally for capability well-being measurement in mental health, using an alternative top-down approach.^6^ This approach, which is rooted in Nussbaum's central human capabilities, is deemed less limited by geographical and cultural contexts.^13^

Empirical comparisons between HRQoL and capability instruments have been conducted in a variety of disease areas, but there is limited information available for mental health conditions.^3^ Furthermore, comparative studies of alternative capability instruments in the area of mental health are lacking, including empirical studies that focus on the use of the ICECAP-A and OxCAP-MH instruments for the same patient population. So far, suggestions for choosing between these measures tended to rest only on the underlying properties of the descriptive and valuation systems.^3^ Therefore, questions remain about how far different applications of the same broad theoretical concept of the capability evaluative space result in different measurement properties, and whether this influences the capability instruments’ properties when compared with HRQoL instruments for optimised research design in mental health. This study aimed to compare the measurement properties of two most commonly used capability instruments in the mental health context, the ICECAP-A and OxCAP-MH,^3^ and compare them to the EQ-5D-5L and the EQ-VAS alongside some mental health-specific instruments in people with schizophrenia experiencing depression.

## Method

### Data

The analysis in this paper was based on data from a study that investigated the impact of positive memory training (PoMeT) on depression symptoms in people with schizophrenia (*n* = 100) in the UK between 2014 and 2016.^14^ Individuals were eligible for inclusion if they were 18–65 years of age, had a DSM-5 diagnosis of schizophrenia or schizoaffective disorder, and had at least a mild level of depression as measured by scoring 14 or more on the Beck Depression Inventory-II (BDI-II).^15^ Participants were identified by trial research assistants who worked in collaboration with care coordinators based in community mental health teams. Participants were assessed at four time points through the 9-month study period: baseline, 3 months, 6 months and 9 months. The assessors, who were masked to treatment allocation, administered the questionnaires to the participants. The PoMeT study was performed in accordance with the Helsinki Declaration and was approved by Berkshire Research Ethics Committee (REC ref. 13/SC/0634). All participants provided written informed consent. More details about the PoMeT trial can be found in Steel et al.^14^

### Instruments

Two capability measures (ICECAP-A and OxCAP-MH), a generic HRQoL instrument (EQ-5D-5L, including the EQ-VAS), and four mental health-specific instruments (Beck Depression Inventory II (BDI-II), Generalized Anxiety Disorder 7 questionnaire (GAD-7), Rosenberg Self-Esteem Scale (RSES) and the Warwick–Edinburgh Mental Wellbeing Scale (WEMWBS)) were used in the study. The focus of this paper is on the comparison of the capability and generic HRQoL instruments with the mental health-specific instruments used as anchors.

#### Capability instruments

##### ICECAP-A

The ICECAP-A is a brief self-reported measure for the general adult population with five items, each of which can take one of four levels, ranging from full capability to no capability. The items include: stability (being able to feel settled and secure), attachment (being able to have love, friendship and support), autonomy (being able to be independent), achievement (being able to achieve and progress) and enjoyment (being able to have enjoyment and pleasure). The ICECAP-A value set used in this study was derived from the UK general population and ranges from 0 (no capability) to 1 (full capability).^16^

The instrument has shown validity,^17^ reliability,^18^ responsiveness^19^ and feasibility^20^ in different populations, including depression,^21^ and it is increasingly used in economic evaluations.^22^

##### OxCAP-MH

The OxCAP-MH is a 16-item self-report instrument developed in the context of mental health outcome measurement. The 16 items cover a broad range of individual capability well-being aspects including: overall health; enjoying social and recreational activities; losing sleep over worry; friendship and support; having suitable accommodation; feeling safe; likelihood of discrimination and assault; freedom of personal and artistic expression; appreciation of nature; self-determination; and access to interesting activities or employment.^4^ The OxCAP-MH items are rated on a 1–5 Likert scale where each question provides an equal contribution to the overall score. The standardised score ranges from 0 to 100, with higher scores reflecting higher levels of capabilities.^23^ The OxCAP-MH has shown validity,^23,24^ responsiveness^23,24^ and feasibility^4^ in several settings and areas of mental illness, including schizophrenia and depression.^25^

#### HRQoL instruments

##### EQ-5D-5L, including EQ-VAS

The EQ-5D instruments are one of the most commonly used self-reported generic health status measures, and their validity and reliability have been reported for various health conditions and populations.^26^ The more recent EQ-5D-5L version comprises five dimensions: mobility, self-care, usual activities, pain/discomfort and anxiety/depression, with 5-level answer options and with preference-based value sets developed in multiple countries.^27^ This study used the UK crosswalk mapping value set developed for the EQ-5D-5L,^28^ following recommendations from the National Institute for Health and Care Excellence.^5^ Scores range between the worst (−0.594) and best (1.000) imaginable health state; zero refers to death. As part of the EQ-5D-5L, respondents’ self-rated health is also recorded on a vertical visual analogue scale (EQ-VAS), where scores range between 0 and 100, referring to worst imaginable health state and best imaginable health state respectively.

#### Mental health-specific instruments

Four mental health-specific instruments used in the PoMeT trial were applied as anchors in the correlation and responsiveness analyses. This was due to their ability to capture the clinically relevant status of the patients together with any changes in status, thereby enabling the responsiveness assessment of the OxCAP-MH, ICECAP-A, EQ-5D-5L and EQ-VAS instruments.

##### BDI-II

The BDI-II is a self-reported measure of depressive symptoms and their severity in adolescents and adults according DSM diagnostic criteria.^15^ It has 21-items, each scored on 4-point polytomous response scale ranging from 0 to 3. Total scores range between 0 and 63, with higher scores representing more severe depression.^15^

##### GAD-7

The GAD-7 is a self-reported measure of anxiety symptoms over the past 2 weeks. It consists of seven items scored on a 0–3 scale, with higher scores indicating more severe symptoms (range from 0 to 21). The cut-off scores of 5, 10 and 15 reflect mild, moderate and severe anxiety symptoms respectively.^29^

##### RSES

The RSES is a 10-item self-report instrument that measures global self-worth by measuring both positive and negative feelings about the self. Items are answered using a 4-point polytomous response scale ranging from strongly agree to strongly disagree. Items 2, 5, 6, 8, 9 are reverse scored. The scale ranges from 0 to 30, with 30 indicating the highest score possible.^30^

##### WEMWBS

The WEMWBS was developed in the UK to assess mental well-being, including affective-emotional aspects, cognitive-evaluative dimensions and psychological functioning. It is a 14-item self-report instrument with 5 response categories (‘none of the time’, ‘rarely’, ‘some of the time’, ‘often’, ‘all of the time’), with a total score ranging from 14 to 70. A higher score indicates a higher level of mental well-being.^31^

### Analyses

We conducted analyses to assess the strength and statistical significance of correlations between instruments using statistical and graphical methods: exploratory factor analysis (EFA) to assess the underlying construct of the measures; assessment of responsiveness, based on Spearman's rank correlations (Spearman's rho) between change scores on the instruments; and agreement analysis using Bland–Altman plots. All analyses were performed using the general population value sets for the ICECAP-A and EQ-5D-5L instruments, with the summed ranges for other instruments. Sensitivity analysis was performed using level sum scores (results are presented in supplementary Appendix 1, available at https://doi.org/10.1192/bjo.2022.514).

For all analyses, the level of significance was set at *P* < 0.05, unless stated otherwise. EFA was conducted using FACTOR 12.01.02 software for Windows (Rovira i Virgili University, Tarragona, Spain; see https://psico.fcep.urv.cat/utilitats/factor/Download.html) and STATA Version 16 for Windows was used for all other analyses. Analysis was conducted on complete cases, excluding missing items at the relevant time point, unless stated otherwise.

#### Convergent validity

We explored convergent validity through correlations across the instruments to test the expectation that capability instruments would be more highly correlated (converge) with each other than with the HRQoL instruments. Convergent validity indicates the degree to which two measures of constructs which are theoretically related are in fact related. Convergence between the instruments was tested through exploring the correlation (Spearman's rank) between their baseline scores and assessed based on Cohen's effect size classification, namely <0.3 is small, 0.3 to <0.5 is moderate and ≥0.5 is large.^32^ Group comparisons of mean baseline scores were conducted using the Wilcoxon rank-sum test^33^ for two-group comparisons and Kruskal–Wallis one-way analysis of variance (ANOVA) for multiple group comparisons.^34^

#### Exploratory factor analysis

We used EFA on the baseline scores of the ICECAP-A, OxCAP-MH and EQ-5D-5L to explore the instruments' factor structure. We conducted EFA for all instruments on the same sample to determine potential overlaps of factors. Making the factor structure explicit contributes to the validity of an instrument.^35^ The factor solution was chosen according to the Kaiser criterion based on a scree plot and the eigenvalues, as described in supplementary Appendix 2, together with further details on the methods of the EFA.

#### Responsiveness

The responsiveness of OxCAP-MH, ICECAP-A, EQ-5D-5L and EQ-VAS was assessed using external anchor instruments. The process started with the definition of two instruments which could be used as autonomous anchors because they identify change that is unlikely to have arisen by chance.^7^ The level of responsiveness was evaluated by defining groups who worsened, improved or remained unchanged, based on whether a clinically meaningful change in instrument scores between baseline and 9-month follow-up assessments was measured for individuals by both anchor instruments. The standard error of measurement (SEM) was calculated based on the difference between the baseline and 9-month values using the following formula:^23,36^
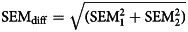


There is no consensus on how many SEMs an individual's score must change by for that change to be considered clinically meaningful. This paper used the threshold of one SEM, which is known frequently to correspond with a minimally important difference.^23,36^ Next, the percentages of the study respondents who improved, worsened or showed no change according to the capability, HRQoL and anchor questionnaires were calculated to explore the detected congruence in changes at the individual patient level. An additional analysis of responsiveness in terms of correlation between change scores (baseline to end-point) of the instruments can be found in supplementary Appendix 5.

#### Agreement analysis

The pattern and extent of agreement between ICECAP-A, OxCAP-MH, EQ-5D-5L and EQ-VAS scores were plotted on Bland–Altman diagrams because they are competitor measures for economic evaluations.^37^ The difference between the instruments is shown on the vertical axis against the mean of the pair on the horizontal axis. For the calculation of the values for the Bland–Altman plots, OxCAP-MH, ICECAP-A, EQ-5D-5L and EQ-VAS scores were standardised to 0–1 intervals where necessary.

## Results

### Participant characteristics

The mean age of participants was 43 years and 75% were males. About half of the participants had received higher education and 86% were unemployed at the time of data collection. Schizophrenia was the primary diagnosis in 70% of the participants, and the rest suffered from schizoaffective disorder or psychosis not otherwise specified. Overall, 83% of participants reported previous hospital admission for psychiatric reasons, with a mean number of 4.5 admissions. The severity of depression was high for 59% of participants. There were significant differences between those with mild to moderate depression and those with depression of high severity in the baseline score for each instrument. Further participant characteristics are presented in Table 1.

**Table 1:** Patient characteristics and mean baseline OxCAP-MH, ICECAP-A, EQ-5D-5L and EQ-VAS scores

	Overall data		OxCAP-MH (0–100)			ICECAP-A (0–1)			EQ-5D-5L (−0.594 to 1)			EQ-VAS (0–100)		
Scale (min–max)	*n*	%	*n*	Mean (s.d.)	*P*^a^	*n*	Mean (s.d.)	*P*^a^	*n*	Mean (s.d.)	*P*^a^	*n*	Mean (s.d.)	*P*^a^
Full cohort	100	100	93	55.66 (12.90)		97	0.525 (0.211)		100	0.534 (0.299)		99	50.14 (21.19)	
Gender														
Male	75	75	69	56.46 (12.25)	0.404	72	0.529 (0.224)	0.486	75	0.548 (0.274)	0.613	74	52.48 (2.39)	0.079
Female	25	25	24	53.39 (14.65)		25	0.513 (0.168)		25	0.515 (0.313)		25	43.20 (4.40)	
Higher education														
Yes	49	49	46	53.36 (1.93)	0.123	47	0.507 (0.201)	0.513	49	0.528 (0.315)	0.909	49	48.91 (3.16)	0.725
No	51	51	47	57.91 (1.81)		50	0.542 (0.220)		51	0.551 (0.252)		51	51.29 (2.89)	
Living situation														
Living with family	19	19	19	51.48 (13.20)	0.440	19	0.488 (0.168)	0.856	19	0.609 (0.199)	0.439	19	57.89 (18.05)	0.263
Renting a flat	12	12	10	59.06 (16.38)		10	0.520 (0.226)		12	0.567 (0.249)		11	46.73 (20.24)	
Owning a flat	5	5	4	51.95 (5.90)		5	0.524 (0.157)		5	0.393 (0.309)		5	43.40 (21.63)	
Other	64	64	60	56.67 (12.41)		63	0.537 (0.226)		64	0.526 (0.307)		64	48.95 (22.03)	
Employment														
Employed full-time	3	3	3	67.19 (14.32)	0.152	3	0.675 (0.122)	0.479	3	0.591 (0.227)	0.004	3	55.00 (18.03)	0.180
Employed part-time	9	9	8	59.18 (9.21)		8	0.580 (0.113)		9	0.589 (0.273)		8	52.13 (20.01)	
Unemployed	86	86	80	55.22 (13.00)		84	0.516 (0.220)		86	0.549 (0.270)		86	50.60 (21.08)	
Other (student/retired)	2	2	2	42.19 (8.84)		2	0.445 (0.100)		2	−0.162 (0.023)		2	15.00 (14.14)	
Primary diagnosis														
Schizophrenia	70	70	66	56.72 (12.77)	0.201	68	0.521 (0.216)	0.859	70	0.545 (0.293)	0.580	70	51.19 (2.48)	0.411
Schizoaffective or psychosis NOS	30	30	27	53.07 (2.52)		29	0.533 (0.201)		30	0.528 (0.263)		29	47.59 (4.15)	
Depression severity														
Mild/moderate	41	41	40	63.44 (1.58)	<0.001	41	0.646 (0.152)	<0.001	41	0.645 (0.238)	0.002	41	56.56 (3.17)	0.011
High	59	59	53	49.80 (1.61)		56	0.436 (0.204)		59	0.467 (0.291)		58	45.59 (2.73)	
Intervention														
Treatment	49	49	46	55.10 (12.59)	0.741	49	0.534 (0.209)	0.634	49	0.571 (0.301)	0.110	49	52.37 (22.82)	0.482
Control group	51	51	47	56.22 (13.30)		48	0.516 (0.213)		51	0.510 (0.264)		50	47.95 (2.75)	

OxCAP-MH, Oxford Capabilities Questionnaire for Mental Health; ICECAP-A, ICECAP capability measure for adults; EQ-VAS, EuroQol Visual Analogue Scale; NOS, not otherwise specified.

a. Wilcoxon rank-sum test for two-group comparison, Kruskal–Wallis one-way ANOVA for multiple group comparison.

### Convergent validity

Mean baseline scores for all instruments and their correlations are presented in Table 2. Graphical presentation of baseline correlations is included in supplementary Appendix 3.

**Table 2:** Baseline scores of the relevant outcome measures used in the trial and the associated Spearman's rank correlations

	Correlation^a^											
	With OxCAP-MH			With ICECAP-A			With EQ-5D-5L			With EQ-VAS		
	*n*	rho	*P*^b^	*n*	rho	*P*^b^	*n*	rho	*P*^b^	*n*	rho	*P*^b^
OxCAP-MH												
ICECAP-A	92	**0.677**	<0.001									
EQ-5D-5L	93	*0.389*	<0.001	97	*0.363*	<0.001						
EQ-VAS	93	*0.354*	0.001	97	*0.431*	<0.001	99	*0.495*	<0.001			
BDI-II	93	−**0.551**	<0.001	97	−**0.505**	<0.001	94	−0.302	0.003	99	−*0.327*	0.001
GAD-7	93	−*0.481*	<0.001	97	−**0.584**	<0.001	94	−**0.518**	<0.001	99	−*0.332*	0.001
RSES	93	−**0.619**	<0.001	97	−**0.558**	<0.001	94	−0.260	0.011	99	−0.234	0.020
WEMWBS	93	**0.718**	<0.001	97	**0.716**	<0.001	94	0.248	0.016	99	*0.377*	<0.001

rho, Spearman's rank correlation coefficient; OxCAP-MH, Oxford Capabilities Questionnaire for Mental Health; ICECAP-A, ICECAP capability measure for adults; EQ-VAS, EuroQol Visual Analogue Scale; BDI-II, Beck Depression Inventory II; GAD-7, General Anxiety Disorder 7; RSES, Rosenberg Self-Esteem Scale; WEMWBS, Warwick–Edinburgh Mental Wellbeing Scale.

a. Moderate correlations (0.3–0.5) in italic, strong correlations (≥0.5) in bold.

b. Wilcoxon matched pairs signed rank test.

Correlations between the capability and HRQoL measures (rho = 0.354–0.431) were lower than those between ICECAP-A and OxCAP-MH (rho = 0.677). The ICECAP-A was slightly more correlated with EQ-VAS (rho = 0.431) than with the EQ-5D-5L (rho = 0.363), whereas the OxCAP-MH was slightly more correlated with the EQ-5D-5L (rho = 0.389) than with the EQ-VAS (rho = 0.354). The baseline scores of both capability instruments were more highly correlated with the mental health-specific instruments (rho = 0.481–0.718) than with the generic HRQoL instruments (rho = 0.354–0.431).

### Exploratory factor analysis

A four-factor solution was selected for EFA. It found that all items of the instruments had commonalities greater than 0.35, i.e. none of the items struggled to load significantly on any factor. Factor loadings are shown in Table 3 for any factor >0.35.

**Table 3:** Exploratory factor analysis of the OxCAP-MH, ICECAP-A and EQ-5D-5L items with four factors using promin rotation (*n* = 78)^a^

	Factor 1	Factor 2	Factor 3	Factor 4
OxCAP-MH				
Limit daily activities	0.453			
Meet socially with friends or family			0.646	
Less sleep over worries		0.523		
Enjoy free-time activities			0.584	
Suitable flat situation	0.391			
Safety in neighbourhood		0.411		
Probability of assault		0.513		
Probability of discrimination		0.635		
Local decisions			0.517	
Freedom of expression			0.384	
Appreciation of nature				0.688
Respect for people around				0.786
Enjoy love and support		0.381	0.562	
Freedom of deciding for yourself		0.510	0.494	
Creativity			0.498	
Access to interesting activities/employment		−0.513	0.752	
ICECAP-A				
Feeling settled and secure		0.499	0.404	
Love, friendship and support			0.725	
Being independent	0.385			
Achievement and progress			0.598	
Enjoyment and pleasure			0.812	
EQ-5D-5L				
Mobility	0.928			
Self-care	0.806			
Daily activities	0.754			
Pain/discomfort	0.761			
Anxiety/depression	0.389			

OxCAP-MH, Oxford Capabilities Questionnaire for Mental Health; ICECAP-A, ICECAP capability measure for adults.

a. Loadings ≤0.35 were removed.

Factor one is an undoubtedly HRQoL-related factor, which consisted of the five EQ-5D-5L and some ICECAP-A and OxCAP-MH items. None of the EQ-5D-5L items loaded on factors two, three and four, thereby suggesting that these factors capture the broader capability-related concepts. Multiple items of the ICECAP-A and OxCAP-MH loaded on these factors, but factor four consisted of only two OxCAP-MH items (‘appreciation of nature’ and ‘respect for people around’), with remarkably high commonalities.

### Responsiveness

The GAD-7 and WEMWBS measures were selected as suitable reference anchor instruments for the analysis of responsiveness since they had the highest correlation with the four scales under investigation in this paper. Table 4 shows the number of participants who improved, showed no change or deteriorated based on the capability, HRQoL and anchor instruments. The OxCAP-MH, ICECAP-A, EQ-5D-5L and EQ-VAS measures identified different proportions of improved (48–52%, 48–52%, 29–35% and 39–52% respectively) and deteriorated (35–53%, 27–35%, 21–33% and 21–33%, respectively) participants in agreement with the anchor instruments. Out of the four scales, the OxCAP-MH identified the largest proportion of participants who had a clinically meaningful change in their capability status over the 9 months (44–54%), and it had the overall best congruency with the anchor measures, especially in terms of identifying deterioration in clinical status (53%), indicating better sensitivity to change than the rest of the measures.

**Table 4:** Number of patients improved, deteriorated or unchanged as defined by the investigated and anchor questionnaires (based on SEM) (*n* = 78)

Instrument	Change^a^	*n*	GAD-7				WEMWBS		
			Improved	No change	Deteriorated	*n*	Improved	No change	Deteriorated

Number of those improved, deteriorated or showed no change	*n*		23	41	14		31	32	15
OxCAP-MH	Improved	24	**12 (52%)**	12 (29%)	0 (0%)	24	**15 (48%)**	9 (28%)	0 (0%)
	No change	38	10 (44%)	**19 (46%)**	9 (64%)	38	13 (42%)	**18 (56%)**	7 (47%)
	Deteriorated	16	1 (4%)	10 (24%)	**5 (35%)**	16	3 (10%)	5 (16%)	**8 (53%)**
ICECAP-A	Improved	22	**12 (52%)**	9 (22%)	1 (7%)	22	**15 (48%)**	4 (13%)	3 (20%)
	No change	44	10 (43%)	**26 (63%)**	8 (57%)	44	13 (42%)	**23 (72%)**	8 (53%)
	Deteriorated	12	1 (4%)	6 (15%)	**5 (35%)**	12	3 (10%)	5 (16%)	**4 (27%)**
EQ-5D-5L descriptive system	Improved	18	**8 (35%)**	8 (20%)	2 (14%)	17	**9 (29%)**	6 (19%)	2 (13%)
	No change	47	14 (61%)	**24 (58%)**	9 (64%)	48	17 (55%)	**23 (72%)**	8 (53%)
	Deteriorated	13	1 (4%)	9 (22%)	**3 (21%)**	13	5 (16%)	3 (9%)	**5 (33%)**
EQ-VAS	Improved	22	**12 (52%)**	6 (15%)	4 (29%)	17	**12 (39%)**	4 (13%)	1 (7%)
	No change	46	11 (48%)	**28 (68%)**	7 (50%)	46	17 (55%)	**20 (62%)**	9 (60%)
	Deteriorated	10	0 (0%)	7 (17%)	**3 (21%)**	15	2 (6%)	8 (25%)	**5 (33%)**

GAD-7, General Anxiety Disorder 7; WEMWBS, Warwick–Edinburgh Mental Wellbeing Scale; OxCAP-MH, Oxford Capabilities Questionnaire for Mental Health; ICECAP-A, ICECAP capability measure for adults; EQ-VAS, EuroQol Visual Analogue Scale.

a. Changes in instrument scores between baseline and 9-month follow-up were categorised as improved, worsened or unchanged; definition of groups is based on the difference in standard error of measurement (SEM); numbers were rounded and do not always add up to 100%; values in agreement are in bold.

### Agreement analysis

**Fig. 1:**
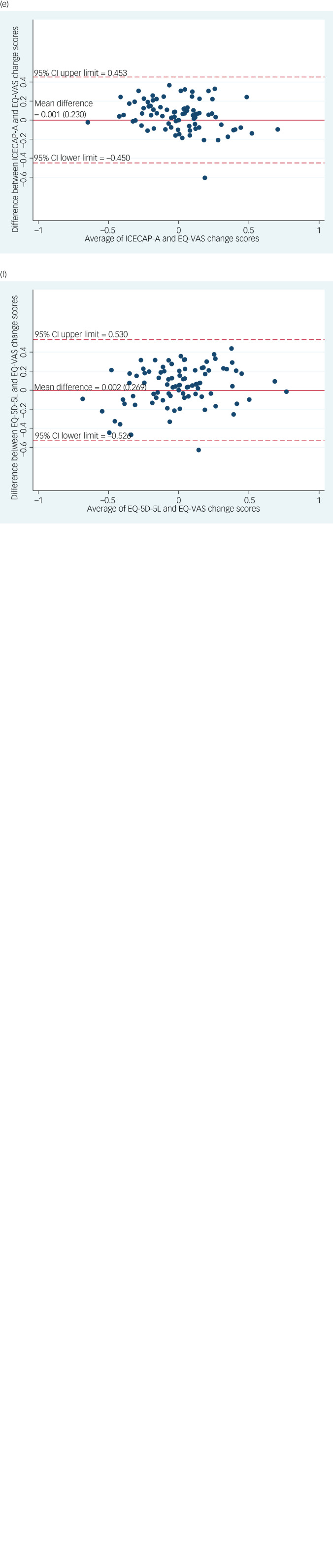
(a) Bland–Altman plot of difference between OxCAP-MH and ICECAP-A change scores (*n* = 78). (b) Bland–Altman plot of difference between OxCAP-MH and EQ-5D-5L descriptive system change scores (*n* = 79). (c) Bland–Altman plot of difference between OxCAP-MH and EQ-VAS change scores (*n* = 79). (d) Bland–Altman plot of difference between ICECAP-A and EQ-5D-5L descriptive system change scores (*n* = 88). (e) Bland–Altman plot of difference between ICECAP-A and EQ-VAS change scores (*n* = 88). (f) Bland-Altman plot of difference of EQ-5D-5L descriptive system and EQ-VAS change scores (*n* = 90). OxCAP-MH, Oxford Capabilities Questionnaire for Mental Health; ICECAP-A, ICECAP capability measure for adults; EQ-VAS, EuroQol Visual Analogue Scale.

The Bland–Altman plots (Fig. 1(a)–(f)) showed that the ICECAP-A and OxCAP-MH have poorer agreement with the EQ-5D-5L than with each other or the EQ-VAS. More specifically, there was small average discrepancy between the four instruments; however, the limits of agreement were wider and therefore more ambiguous in the comparisons with the EQ-5D-5L descriptive system than in the direct comparison of the OxCAP-MH, ICECAP-A and EQ-VAS.

## Discussion

### Summary and interpretation of the findings

This paper aimed to contribute to the utilisation of the capability approach in the context of mental health by assessing how far alternative capability instruments capture broader outcome information in people with schizophrenia and depression compared with the HRQoL and in relation to each other, with some mental health-specific measures serving as anchors. At baseline, the two capability instruments were strongly correlated with each other, but moderately correlated with the EQ-5D-5L and EQ-VAS. Both capability measures correlated with mental health-specific instruments more strongly than the two HRQoL measures and proved superior to the HRQoL scales in terms of responsiveness. Together with the results of the EFA and the Bland–Altman plots, our findings confirm that the capability evaluative space captures aspects of what might be important for individuals with severe mental health conditions beyond the aspects that commonly used HRQoL scales are able to measure. This is also suggestive of their ability to measure important effects of mental health interventions beyond health. In general, our research shows that capability instruments may be seen as complementary to HRQoL instruments in their measurement properties in this context.

This paper also compared the psychometric properties of the two capability instruments with each other in the context of the given severe mental health condition. The study empirically demonstrated that these two instruments show somewhat different psychometric properties when deployed on the same patient cohort despite their common theoretical framework. The ICECAP-A showed a slightly weaker anchor-based responsiveness to depression, anxiety and mental well-being as measured by the GAD-7 and WEMWBS scales than the OxCAP-MH. Furthermore, the EFA and responsiveness analyses suggested a somewhat broader evaluative space and better sensitivity to change for the OxCAP-MH in comparison with the ICECAP-A. These differences may be explained by: (a) their different approaches to development, (b) the number of items in the measures (5 items in the ICECAP-A, 16 items in the OxCAP-MH) and (c) the more mental health-specific nature of the OxCAP-MH compared with the fully generic nature of the ICECAP-A.

### Relationship between the findings and the existing literature

This analysis found slightly weaker correlations between the capability instruments and the EQ-5D-5L than some of the previous studies conducted in the area of mental health. The OxCAP-MH was compared with the EQ-5D-3L and EQ-5D-5L in mixed mental health populations and found correlation coefficients between 0.45 and 0.66.^23,24^ Similar correlations were observed between the ICECAP-A and the EQ-5D when they were compared for opiate-dependent individuals: one study found that the ICECAP-A and EQ-5D-5L have similar construct validity when compared with other clinical measures.^38^ The slightly different results of the current study seem confirmatory of the previously identified weaknesses of the EQ-5D-5L instrument in measuring HRQoL in people with severe or complex mental illness.^39^ Our results are also similar to the findings of a study comparing the ICECAP-A and EQ-5D-5L in the area of depression, which concluded that instruments designed specifically to measure depression and mental health explained a greater proportion of the variation in the ICECAP-A than in the EQ-5D-5L.^21^ The slightly weaker correlations between the ICECAP-A and EQ-5D-5L could be explained by the specific area of depression, where evidence has shown that this disease is associated with a higher level of disability on the item of interpersonal activities.^40^ Moreover, previous factor analyses comparing the ICECAP-A with the items of the EQ-5D-5L and EQ-5D-3L found that these instruments measure two different constructs and therefore provide potentially different information. A recent systematic literature review found inconsistencies between the ICECAP-A and EQ-5D instruments, suggesting that the ICECAP-A is most appropriately regarded as a complement to and not a substitute for the EQ-5D-3L and EQ-5D-5L.^36^

For the OxCAP-MH, a recent study found that the capability measure had strong correlations with the EQ-5D-5L, EQ-VAS and mental health-specific instruments.^23,24^ This indicates that the OxCAP-MH indeed measures some aspects of HRQoL and can be considered enhanced rather than fully complementary to the EQ-5D-5L.^24^ The same study deployed a two-factor EFA and found that all EQ-5D-5L items and seven OxCAP-MH items loaded on one factor, while nine remaining OxCAP-MH items loaded on a separate factor, also suggestive of the enhanced nature of the instrument when compared with the EQ-5D-5L. The results of the responsiveness analyses from the current study and those from Łaszewska et al^24^ are also in line, both concluding that the OxCAP-MH is responsive to the changes in individuals’ health states over time measured by anchor questionnaires, and it discriminates between defined patient groups with high sensitivity.

### Strengths and limitations

The study benefited from a data-set that enabled comparison between the two most commonly used capability instruments in the area of mental health and generic HRQoL and mental health-specific scales, and comparison between the two capability instruments themselves. The scope of the paper included various aspects of mental well-being and provided the opportunity for a comprehensive investigation of the capability instruments’ measurement properties in the same context and under the same conditions.

Limitations of this research included a restricted number of data points compared with the number of items, which might affect the robustness of the EFA and the responsiveness analyses, the two methods most sensitive to sample size problems. The study used value sets for the ICECAP-A and EQ-5D-5L but not for the OxCAP-MH, which can introduce an exogenous source of variance into statistical inference. Nevertheless, the sensitivity analysis reported in supplementary Appendix 1 confirmed that this had an insignificant effect on the findings of this study. Finally, the study conducted the comparison and established the psychometric properties of the instruments in a specific context; hence, the results should be generalised with caution to other diseases.

### Implications for practice/policy

To our knowledge, this is the first study to empirically compare the two most commonly used capability instruments in the area of mental health and compare them simultaneously to HRQoL and mental health-specific scales. The findings of this study provide an in-depth analysis and understanding of how the method of operationalising the capability approach and the items included in the instruments may eventually influence the interpretation of an economic evaluation based on these instruments.

Our findings confirmed that capability instruments capture complementary information compared with HRQoL instruments. Therefore, consideration needs to be given to the inclusion of a valid capability instrument in future research studies in the area of (severe) mental illness. This is particularly relevant for economic evaluations that are conducted from a societal perspective, where outcomes beyond health play an important role. Currently, a context-specific choice between the ICECAP-A and OxCAP-MH should be based on the trade-off between the higher responsiveness and broader evaluative space of the OxCAP-MH against the somewhat lower participant burden and the currently available value set for the ICECAP-A.

### Implications for future research

Establishing the psychometric properties of an instrument is context specific; therefore, these conclusions must be strengthened in other disease areas. Furthermore, comparisons of the two investigated capability instruments with other, newer well-being ones developed for the area of mental health, such as the Achieved Capabilities Questionnaire for Community Mental Health (ACQ-CMH) or Recovering Quality of Life (e.g. ReQol), would further contribute to our understanding of their comparative measurement characteristics and potentially differing outcome results if measured simultaneously. Future research should explore further the effect of value-based scoring once this also becomes available for the OxCAP-MH instrument.

## Data Availability

The data that support the findings of this study are available on request from the corresponding author.

## Supplementary material

For supplementary material accompanying this paper visit http://doi.org/10.1192/bjo.2022.514. click here to view supplementary material
