# Supplementary material for: Comparison of capability and health-related quality of life instruments in capturing aspects of mental well-being in people with schizophrenia and depression
Source: BJPsych Open. 2022 Jun 27;8(4):e117. doi: 10.1192/bjo.2022.514 (PMC9301777; doi:10.1192/bjo.2022.514)
Supplement: Supplementary file 1 [file S2056472422005142sup001.pdf]

Contents of the supplementary material

Appendix 1: Sensitivity analysis using level sum scores 2
Appendix 2: Further details on the Exploratory Factor Analysis 5
Appendix 3: Graphical presentation of correlations between baseline scores 8
Appendix 4: Assessing responsiveness using external anchor instruments 11
Appendix 5: Assessing responsiveness in terms of correlation between change scores 13
References 15

Appendix 1: Sensitivity analysis using level sum scores

Objective

To provide a sensitivity analysis using level sum scores to analysis of correlations between baseline and change scores.

Methods

The analysis of correlations between baseline and change scores were repeated using level sum scores instead of preference-based scores for the ICECAP-A and EQ-5D-5L instruments. It means that the relevant parts of Tables 1, 2 and 4 were recalculated.

Applying value sets is an important aspect in case of economic evaluations; however, it can also introduce an exogenous source of variance into statistical inference (1). Moreover, relevant tariff values for the EQ-5D-5L descriptive system and the ICECAP-A have different anchor points. The 0 point of the EQ-5D-5L value set is anchored against 'death', while the 0 point of the ICECAP-A value set is anchored against 'no capability' leading to potential difficulties in interpreting in any comparisons based on preference-weighted scales. Hence, a sensitivity analysis was performed to the analysis conducted with preference-based weights for the EQ-5D-5L and the ICECAP-A instruments.

Results

The results of the analyses based on level sum scores did not significantly differ from those performed with preference-based scores.

Table S1: Patient characteristics and mean baseline OxCAP-MH, ICECAP-A, EQ-5D-5L and EQ VAS scores based on level sum scores

**Scale (Min-Max)**		**ICECAP-A (5-20)**				**EQ-5D-5L descriptive system (5-25)**			
		n	Mean baseline score (SD)	0-1 score*	*p***	n	Mean baseline score (SD)	0-1 score*	*p***
**Full cohort**		97	11.35 (2.86)	0.42		100	18.73 (4.03)	0.68	
**Gender**	Male	72	11.43 (3.03)	0.43	0.642	75	18.89 (0.45)	0.69	0.486
	Female	25	11.12 (2.32)	0.41		25	18.24 (0.88)	0.66	
**Higher education**	Yes	47	11.04 (2.63)	0.40	0.306	49	18.45 (0.63)	0.67	0.497
	No	50	11.64 (3.06)	0.44		51	19.00 (0.51)	0.70	
**Living situation**	Living with family	19	10.84 (2.27)	0.39	0.799	19	19.58 (2.91)	0.73	0.506
	Renting a flat	10	11.20 (2.86)	0.41		12	19.00 (3.59)	0.70	
	Owning a flat	5	11.00 (1.73)	0.40		5	16.60 (4.22)	0.58	
	Other	63	11.56 (3.10)	0.44		64	18.59 (4.37)	0.68	
**Employment**	Employed full-time	3	13.33 (1.53)	0.56	0.609	3	19.00 (3.46)	0.70	0.106
	Employed part-time	8	11.75 (1.39)	0.45		9	19.44 (4.00)	0.72	
	Unemployed	84	11.26 (3.01)	0.42		86	18.86 (3.88)	0.69	
	Other (Student/Retired)	2	10.50 (0.71)	0.37		2	9.50 (0.71)	0.23	
**Primary diagnosis**	Schizophrenia	68	11.34 (2.89)	0.42	0.949	70	18.84 (0.50)	0.69	0.671
	Schizoaffective or psychosis NOS***	29	11.38 (2.81)	0.43		30	18.47 (0.69)	0.67	
**Depression severity**	Mild/moderate	41	12.88 (2.25)	0.53	0.000	41	20.07 (0.58)	0.75	0.005
	High	56	10.23 (2.75)	0.35		59	17.80 (0.52)	0.64	
**Intervention**	Treatment	49	11.33 (2.76)	0.42	0.934	51	18.24 (0.53)	0.66	0.212
	Control group	48	11.36 (2.99)	0.42		49	19.25 (0.61)	0.71	

**scores were standardised to a 0-1 range for all instruments for reasons of comparability*

**Wilcoxon rank-sum test for two-group comparison, Kruskal–Wallis one-way ANOVA for multiple group comparison*

****NOS: Not Otherwise Specified*

Table S2: Baseline scores of the relevant outcome measures used in the trial and the associated Spearman's rank correlations based on level sum scores

	with OxCAP-MH			with ICECAP-A			with EQ-5D-5L			with EQ VAS		
	n	Coef.	p value*	n	Coef.	p value*	n	Coef.	p value*	n	Coef.	p value*
OxCAP-MH												
ICECAP-A	92	**0.682**	0.000									
EQ-5D-5L	93	*0.381*	0.000	97	*0.344*	0.001						
EQ VAS	93	*0.354*	0.001	97	*0.425*	0.000	99	*0.479*	0.000			
BDI	93	**-0.551**	0.000	97	**-0.511**	0.000	100	-0.286	0.004	99	*-0.327*	0.001
GAD	93	*-0.481*	0.000	97	**-0.575**	0.000	100	*-0.487*	0.000	99	*-0.332*	0.001
RSES	93	**-0.619**	0.000	97	**-0.570**	0.000	100	-0.243	0.015	99	-0.234	0.020
WEMWBS	93	**0.718**	0.000	97	**0.710**	0.000	100	0.208	0.038	99	*0.377*	0.000

BDI: Beck Depression Inventory; GAD: General Anxiety Disorder; RSES: Rosenberg Self-Esteem Scale; WEMWBS: Warwick-Edinburgh Mental Well-Being Scale; Moderate correlations (0.3-0.5) in italic, **Strong correlations (>=0.5) in bold;** **Wilcoxon matched pairs signed rank test*

Appendix 2: Further details on the Exploratory Factor Analysis

Objective

To provide further details on the Exploratory Factor Analysis (EFA), including the testing of data for suitability, choice of the number of factors retained, main analysis method and rotation of factor loadings.

Methods

Prior to EFA, data were tested for suitability for factor analysis by Kaiser-Meyer Olkin (KMO) and Bartlett's tests (2). KMO of sampling adequacy is a statistic that indicates the proportion of variance in the included variables that might be caused by underlying factors. Good suitability for factor analysis is indicated by a bare minimum of 0.5 (3) and the value between 0.5 and 0.7 are mediocre, value between 0.7 and 0.8 are good, value between 0.8 and 0.9 are great and value between 0.9 and above are superb (4). The strength of the relationship among variables was confirmed by a statistically significant Bartlett‘s Test, which verifies the assumption that variances are equal across groups or samples (2). Good internal consistency was determined by a Cronbach's alpha greater than 0.70 (4). Factor analysis was performed using polychoric correlations adequate for categorical scales using the freely available FACTOR software. The number of factors retained was chosen according to the Kaisers criterion based on a scree plot and the eigenvalues. Minimum Rank Factor Analysis (4) was chosen as the main analysis method. Factor loadings were rotated using promin rotation because it is known to perform better than other well-known oblique rotation procedures (5, 6). The correlation between factors is provided on Figure S8.

Figure S4: Scree plot of eigenvalues (OxCAP-MH, ICECAP-A, EQ-5D-5L)

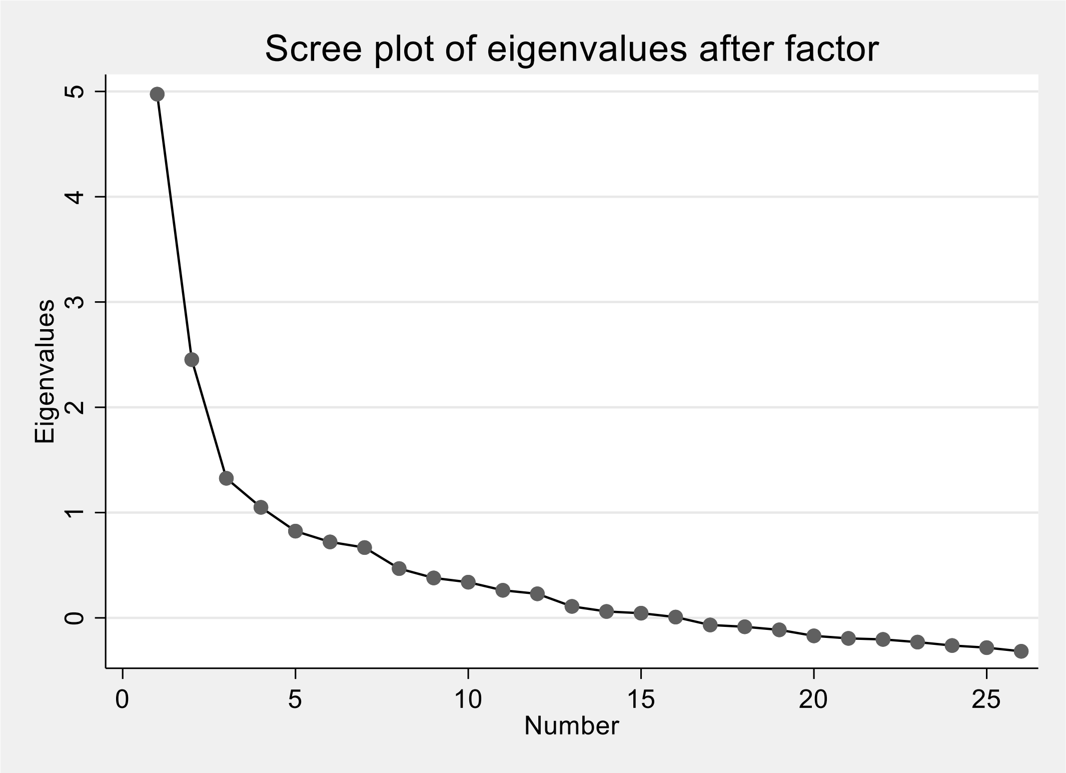

Figure S5: List of Eigenvalues

```
Factor analysis/correlation
    Method: principal factors
    Rotation: (unrotated)
```

Factor	Eigenvalue
Factor1	4.97437
Factor2	2.45269
Factor3	1.32585
Factor4	1.04996
Factor5	0.82398
Factor6	0.72157
Factor7	0.66769

Figure S6: Inter-factor correlation matrix

Factor	F 1	F 2	F 3	F 4
1	1.000			
2	0.385	1.000		
3	0.194	0.180	1.000	
4	0.127	0.045	0.120	1.000

Results

The Kaiser–Meyer–Olkin test of sampling adequacy resulted in a fair value of 0.724 (CI: 0.724-0.758) and Bartlett's test of sphericity resulting in a c2 of 785.8, (p = 0.00001), suggesting that the sample is suitable for factor analysis. Cronbach's alpha for all 26 items was 0.8416, suggesting that the three instruments together possess a reliable internal consistency. Figure 5 shows the scree plot of eigenvalues based on the three instruments, and Figure S3 (List of Eigenvalues) presents them in a tabular format. Since the dots in Figure S2 (Scree plot of eigenvalues) are very close to the threshold of 1, the decision as to the number of factors was based on the numerical format of Eigenvalues. The combined information supported a four-factor solution for eigenvalues >1. Figurer S4 shows that the inter-factor correlations are very low.

Appendix 3: Graphical presentation of correlations between baseline scores

Objective

To provide a graphical presentation of correlations between baseline scores of the scales under investigation, including OxCAP-MH, ICECAP-A, EQ-5D-5L and EQ VAS.

Methods

Scores of respondents on the relevant scales were plotted on scatterplots. Graphical presentation of correlation between baseline and change scores explored the degree of agreement between the four scales. The axis of the graphs represented the minimum and maximum values of the relevant instruments.

Results

The findings echo those presented in Table 2 of the main paper.

Figure S7: Correlations between OxCAP-MH and ICEAP-A scores at baseline (n=92)

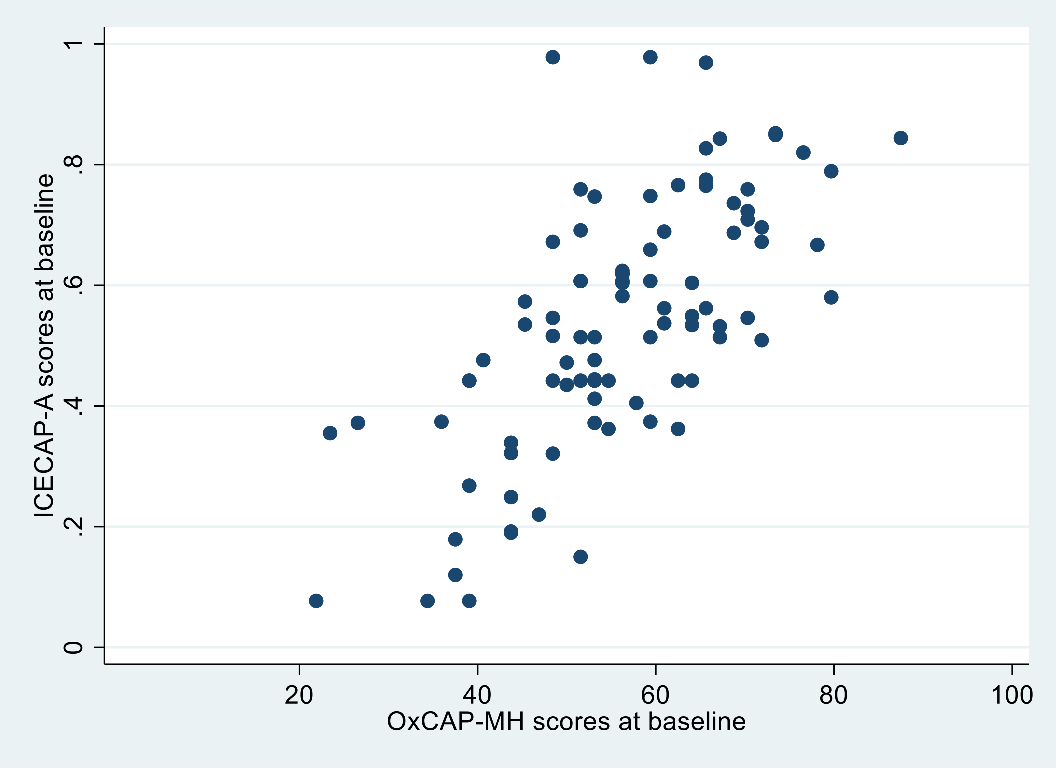

Figure S8: Correlations between OxCAP-MH and EQ-5D-5L scores at baseline (n=93)

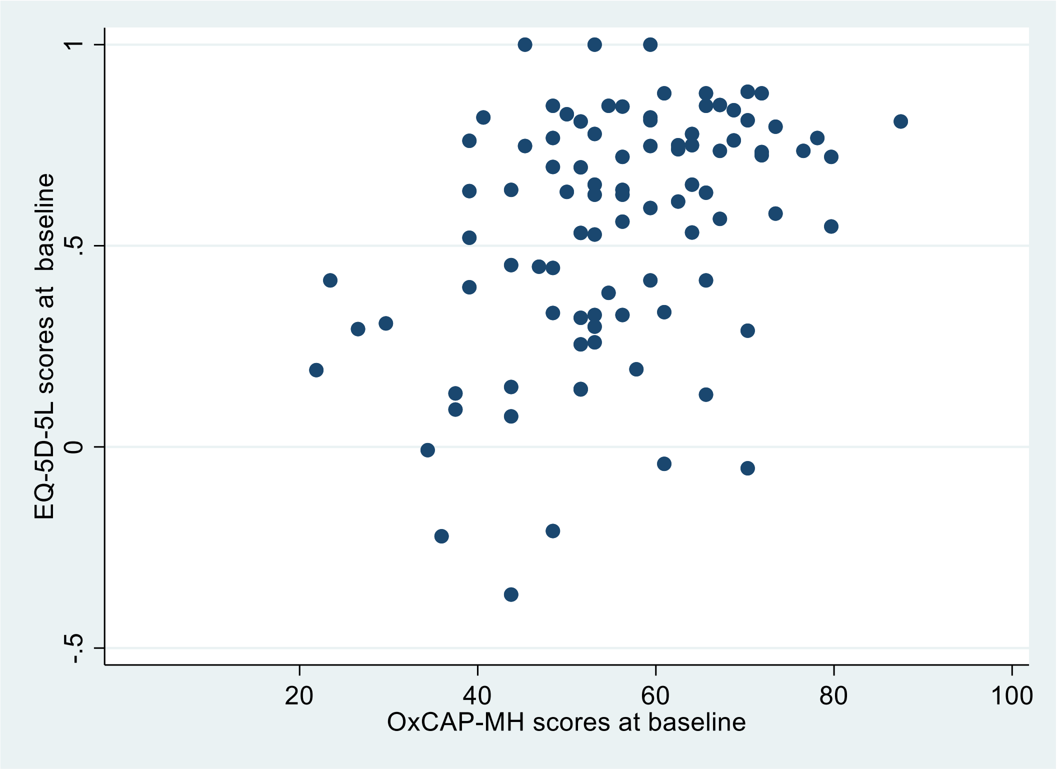

Figure S9: Correlations between OxCAP-MH and EQ VAS scores at baseline (n=93)

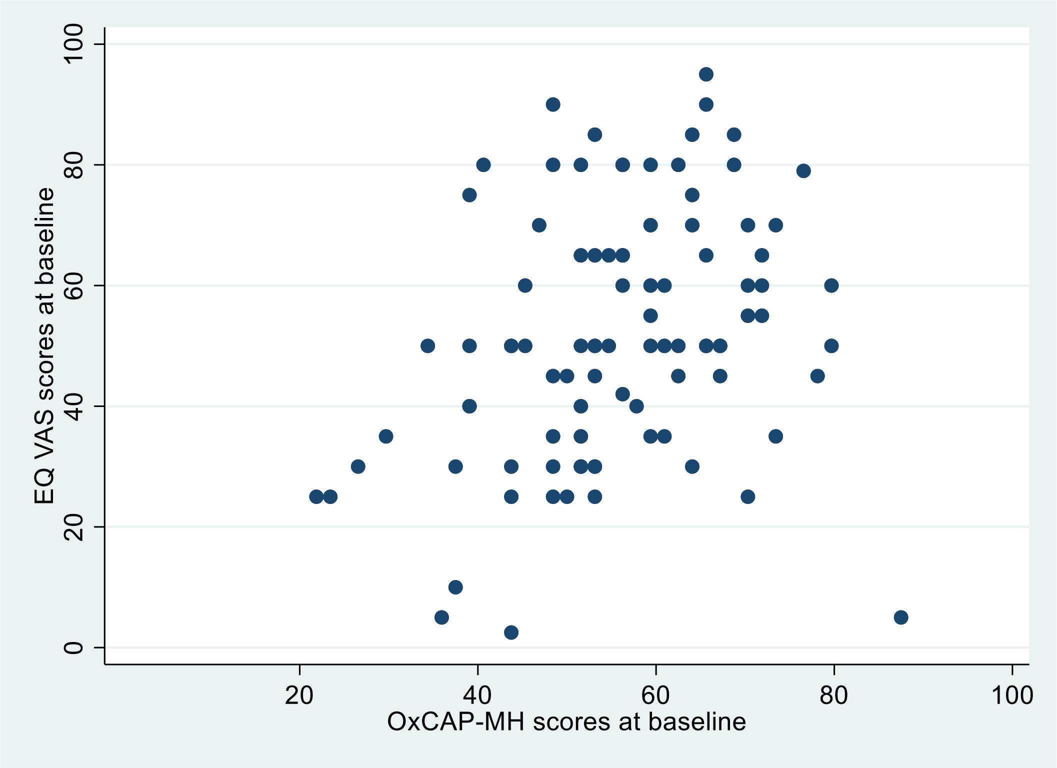

Figure S10: Correlations between ICECAP-A and EQ-5D-5L scores at baseline (n=97)

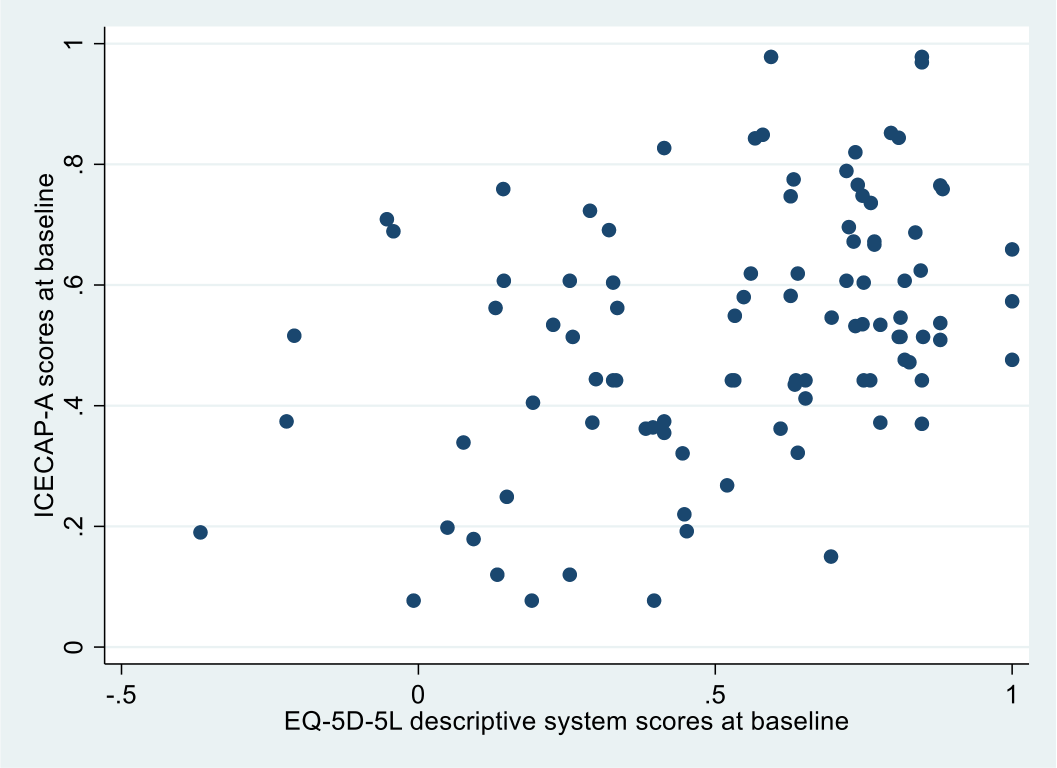

Figure S11: Correlations between ICECAP-A and EQ VAS scores at baseline (n=97)

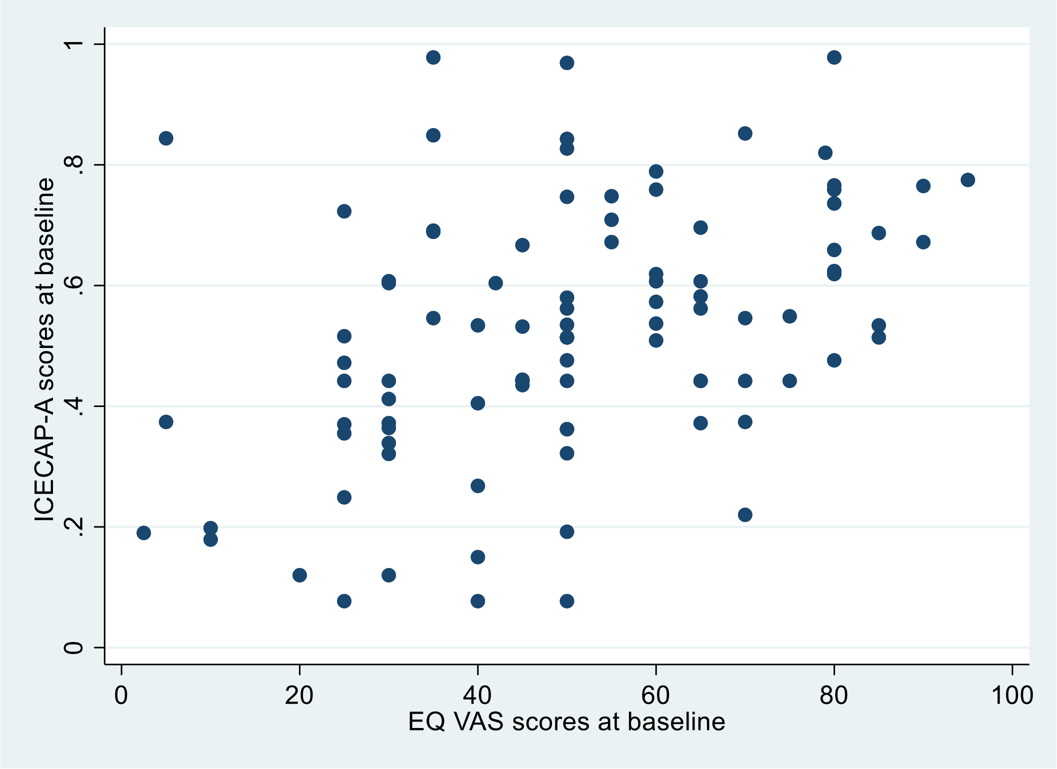

Figure S12: Correlations between EQ-5D-5L and EQ VAS scores at baseline (n=99)

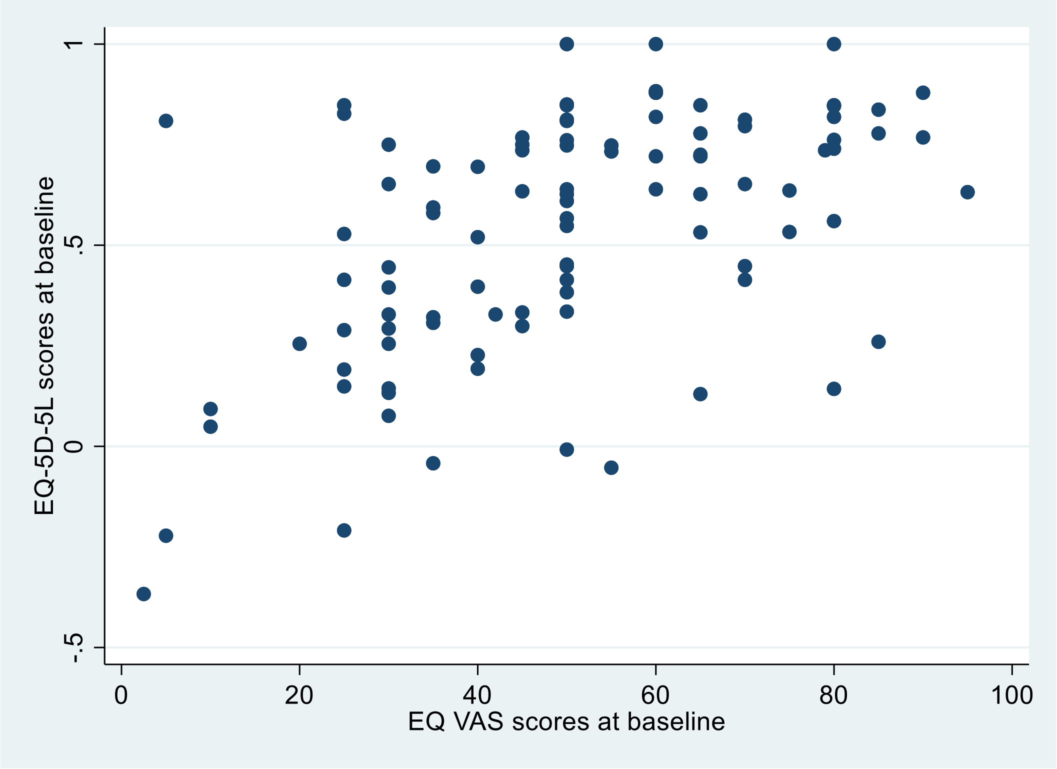

Appendix 4: Assessing responsiveness using external anchor instruments

Objective

To provide further details about the SEM-based analysis, including a more detailed formula and the interim results of the calculation.

$$SEM_{diff} = \sqrt{\left(SD_{baseline} * \sqrt{1 - Alpha_{baseline-3\ months}}\right)^2 + \left(SD_{9\ months} * \sqrt{1 - Alpha_{6\ months-9\ months}}\right)^2}$$

Table S13: Calculation of SEM for each instrument (n=78)

	OxCAP-MH	ICECAP-A	EQ-5D-5L descriptive system	EQ VAS	GAD	WEMWBS
SEM Baseline	5.16	0.099	0.123	14.06	2.64	4.07
SEM 9 months	4.62	0.081	0.113	12.70	2.66	4.29
SEM_{diff}	6.93	0.128	0.167	18.94	3.75	5.92

Appendix 5: Assessing responsiveness in terms of correlation between change scores

Objective

To assess the responsiveness of OxCAP-MH, ICECAP-A, EQ-5D-5L and EQ VAS scales in terms of correlation between change scores (baseline to endpoint) of the instruments.

Methods

Patients filled out each scale at both baseline and 9 months, which allowed for an exploration of change in mean scores over time across all instruments. Responsiveness was defined as the ability to capture change over time and was assessed by Spearman's rank correlation between baseline to endpoint change scores (7).

The Spearman's rank correlations between the baseline to endpoint change scores of the ICECAP-A, OxCAP-MH, EQ-5D-5L, EQ VAS and the potential reference instruments are presented in Table S14.

Results

Correlation between the ICECAP-A and OxCAP-MH change scores was moderate (0.388). The ICECAP-A change scores weakly correlated with the HRQoL change scores, i.e. the EQ-5D-5L and EQ VAS (0.255-0.269), whilst moderate correlations were observed with the mental health-specific instruments (0.355-0.451). The OxCAP-MH change scores also had weak correlations with the HRQoL change scores (0.200-0.203), whilst moderate to high correlations with the mental health-specific instruments (0.440-0.557).

Table S14: Spearman's rank correlations between change scores of OxCAP-MH, ICECAP-A, EQ-5D-5L, EQ VAS and mental health specific instrument scores

	**OxCAP-MH**			**ICECAP-A**			**EQ-5D-5L**			**EQ VAS**		
	n	Coef	p value*	n	Coef	p value*	n	Coef	p value*	n	Coef	p value*
OxCAP-MH												
ICECAP-A	78	*0.388*	<0.001									
EQ-5D-5L	79	0.200	0.078	88	0.255	0.017						
EQ VAS	79	0.203	0.391	88	0.269	0.011	90	*0.413*	<0.001			
Beck Depression Inventory (BDI)	79	*-0.437*	<0.001	88	*-0.355*	0.001	94	-0.195	0.056	90	-0.198	<0.001
Generalized Anxiety Disorder scale (GAD)	79	**-0.557**	0.065	88	*-0.451*	<0.001	92	-0.215	0.040	90	-0.272	0.015
Rosenberg Self-Esteem Scale (RSES)	79	*-0.440*	0.006	87	*-0.387*	<0.001	91	-0.244	0.020	89	-0.250	0.007
Warwick-Edinburgh Mental Wellbeing Scale (WEMWBS)	79	*0.493*	0.837	88	*0.385*	<0.001	91	0.217	0.039	89	0.270	0.264

Moderate correlations (0.3-0.5) in italic, **Strong correlations (>=0.5) in bold;** **Wilcoxon matched pairs signed rank test*

References

1. Parkin D, Rice N, Devlin N. Statistical analysis of EQ-5D profiles: does the use of value sets bias inference? Med Decis Making. 2010;30(5):556-65.
2. Bartlett MS. A note on multiplying factors for various chi-squared approximations. Joural of the Royal Statistical Society, Series B. 1954;16:296–8.
3. Terwee CB, Dekker FW, Wiersinga WM, Prummel MF, Bossuyt PMM. On assessing responsiveness of health-related quality of life instruments: Guidelines for instrument evaluation. Quality of Life Research. 2003;12(4):349-62.
4. Shapiro A, ten Berge JMF. Statistical inference of minimum rank factor analysis. Psychometrika. 2002;67(1):79-94.
5. Lorenzo-Seva U. Why rotate my data using Promin? Technical report. Tarragona; 2013.
6. Lorenzo-Seva U. Promin: A Method for Oblique Factor Rotation. Multivariate Behavioral Research. 1999;34(3):347-65.
7. Husted JA, Cook RJ, Farewell VT, Gladman DD. Methods for assessing responsiveness: a critical review and recommendations. Journal of Clinical Epidemiology. 2000;53(5):459-68.
